# Supplementary material for: Barriers against Implementation of European Society of Gastrointestinal Endoscopy Performance Measures for Colonoscopy in Clinical Practice
Source: Medicina (Kaunas). 2024 Jul 19;60(7):1166. doi: 10.3390/medicina60071166 (PMC11279203; doi:10.3390/medicina60071166)
Supplement: Supplementary file 1 [file medicina-60-01166-s001.zip › medicina-3074693-supplementary.pdf]

SECTION I

DEMOGRAPHIC AND PROFESSIONAL CHARACTERISTICS

1. Gender

a. Male

b. Female

2. Age: ________________________________

3. Year of training in Gastroenterology or Digestive Endoscopy:_______________

4. Area of practice

a. North-West (Valle d'Aosta, Piemonte, Lombardia, Liguria)

b. North-East (Friuli-Venezia Giulia, Veneto, Emilia Romagna, Trento/Bolzano)

c. Centre (Toscana, Marche, Umbria, Lazio)

d. South and Islands (Abruzzo, Molise, Campania, Puglia, Basilicata, Calabria, Sicilia, Sardegna)

5. What is your working environment?

a. Community hospital

b. Academic hospital

c. Private hospital/centre or contracted private hospital/centre

6. Is your Centre a CCR Screening Centre with dedicated sessions?

a. No

b. Yes

7. What year does your endoscopy reporting system date back to? ______________________

8. What are the make and model of the endoscopic instruments you use in your Centre?

____

SECTION II

CULTURAL DETAILS

1. Does your Centre organise periodic audits to monitor the level of quality offered by Endoscopists?

a. No

b. Yes

2. Do you participate (e.g. 1 time every 2-3 years) in courses or events on the quality in digestive endoscopy?

a. No

b. Yes

3. Do you know what quality indicators for colonoscopy are proposed by ESGE and implemented by SIED?

a. No

b. Yes

4. Does your reporting system allow automated extraction of the quality indicators proposed by ESGE?

a. Yes

b. No

SEZIONE II

PRE-PROCEDURE ASSESSMENT

1. What percentage of diagnostic colonoscopies do you disagree with in a session?

a. <5% (STANDARD TARGET)

b. 5-15% (MINIMUM STANDARD)

c. 10-25%

d. 25-50%

e. >50%

2. How do you deal with inappropriate requests for colonoscopy?

a. I perform it

b. I do not perform it

c. I discuss the indication with the requesting physician and agree on how to act

d. I discuss the indication with the patient and agree how to act

3. Do you carry out periodically in a structured manner an evaluation of the appropriateness of the colonoscopies requested by GPs using the ESGE performance indicators?

a. No

b. Yes

4. Does your reporting system provide for a structured definition of the appropriateness of the indication for endoscopic exam?

a. No

b. Yes, according to EPAGE-II criteria

c. Yes, according to ASGE criteria

d. Yes, according to RAO criteria

e. Yes, other (specify) ____

5. Does your reporting system allow you to automatically extract the percentage of colonoscopies with adequate toilette in your centre?

a. No

b. Yes, please specify the percentage ________________

6. Does your reporting system structurally provide for the inclusion of a validated bowel preparation scale?

a. Yes, BBPS

b. Yes, Aronchick

c. Yes, Ottawa

d. Yes, Harefield

e. No

7. Does your reporting system structurally provide for the entry of the type of bowel preparation taken by the patient?

a. No

b. Yes

8. What is the bowel preparation used by your centre for screening for CCR ?

a. PEG-4L

b. PEG-2L + citrate

c. PEG-2L + ascorbate

d. PEG-1L + ascorbate

e. Other (specify) ___________________________

f. My centre does not provide bowel preparation for screening

9. What preparation regimen does your centre recommend for patients undergoing colonoscopy as part of CCR screening?

a. Split (half/three-quarters the day before and half/one-quarter the day of the examination)

b. All on the day of the examination

c. All on the day before the exam

d. My centre does not provide guidance on the intake regime to be followed

10. The preparation administered in split-dose regimen:

a. Has no significant effect on the main indicators of colonoscopy quality

b. Improves patient tolerability, but does not impact on the quality of intestinal cleansing

intestinal cleansing

c. Improves quality of bowel cleansing, but has no impact on ADR

d. Improves quality of bowel cleansing and increases ADR

11. Does your reporting system structurally include the mode of bowel preparation (e.g. split-dose)?

a. No

b. Yes

12. What is the time interval to be observed between the end of taking the preparation

intestinal preparation and the start of the examination, in order to improve the effectiveness of the preparation?

a. Between 2 and 5 hours

b. At least 5 hours

c. The longer the time, the better the effect of bowel preparation

d. The effectiveness of the preparation is independent of the time interval between the end

intake and start of the examination

13. Does your reporting system provide for the input of the interval between the end of preparation and start of examination?

a. No

b. Yes

14. What is in your perception the main factor responsible for inadequate bowel preparation?

a. Absence of simethicone

b. Low volume preparation

c. Preparation at very low volume

d. Patient characteristics (e.g. chronic constipation, hospitalisation, adherence syndrome)

e. Insufficient intake of preparation

f. Intake of preparation in an incongruous manner (e.g. all the day before)

15. What in your perception is the main factor(s) responsible for insufficient intake of preparation?

a. Bad taste

b. Induction of vomiting

c. Too high volume

d. Interference with normal activities (e.g. work)

e. Insufficient patient motivation/information

16. In terms of acceptability of the preparation and patients willing to repeat the preparation what is the difference between 2L and 1L PEG-based solutions?

a. More patients are willing to repeat the preparation with solutions based on

PEG 2L-based solutions therefore has a higher acceptability by patients

b. Are more patients willing to repeat the preparation with solutions based on

PEG 1L solutions, thus presenting greater acceptability by patients

c. There is no difference in acceptability between the two solutions

17. Who provides the bowel preparation for colonoscopy in your centre has received specific training?

a. No

b. Yes

18. Does your reporting system report in a structured manner (e.g. checklist) the achievement of the caecal fundus?

a. No

b. Yes

19. Does your reporting system allow you to extrapolate your ADR, integrating endoscopic examination data with the pathology service?

a. No

b. Yes, ___________________(specify)

20. Do you independently monitor your ADR?

a. No

b. Yes, _________________(specify)

21. Does your reporting system allow you to indicate in a structured manner the technique of polypectomy according to the size of the lesions removed?

a. No

b. Yes

SECTION III

POST-PROCEDURAL ASPECTS

1. Does your reporting system allow you to automatically extract the performance indicators according to ESGE for colonoscopy?

a. No

b. Yes

2. Does your reporting system provide for structured monitoring of the level of satisfaction of patients towards bowel preparation?

a. No

b. Yes, it is >90%

c. Yes, it is 75-90%

d. Yes, it is 50-75%

e. Yes, it is <50%

3. Does your reporting system provide structured monitoring of the level of satisfaction of patients for the "global" colonoscopy experience, i.e. from when they enter to when they they leave the Digestive Endoscopy Service?

a. No

b. Yes, it is >90%

c. Yes, it is 75-90%

d. Yes, it is 50-75%

e. Yes, it is <50%

4. Does your endoscopy service provide systematic monitoring of late complications post colonoscopy?

a. No

b. Yes

5. Does your reporting system structurally provide for the possibility of providing written indications regarding follow-up?

a. No

b. Yes
